# Bridging the microscopic divide: a comprehensive overview of micro-crystallization and *in vivo* crystallography

**DOI:** 10.1107/S205225252400513X

**Published:** 2024-06-27

**Authors:** Leonard Michel Gabriel Chavas, Fasséli Coulibaly, Damià Garriga

**Affiliations:** a NUSR Nagoya University Nagoya Japan; b Applied Physics Nagoya University Nagoya Japan; c Biomedicine Discovery Institute & Department of Biochemistry and Molecular Biology Monash University Clayton Australia; d ALBA Synchrotron Cerdanyola del Vallès Spain; University of Auckland, New Zealand

**Keywords:** micro-crystallization, *in vivo* crystallography, structural biology, macromolecular research, *in situ*X-ray diffraction, XFELs, MicroED

## Abstract

A series of events underscoring the significant advancements in micro-crystallization and *in vivo* crystallography were held during the 26th IUCr Congress in Melbourne, positioning microcrystallography as a pivotal field within structural biology. Through collaborative discussions and the sharing of innovative methodologies, these sessions outlined frontier approaches in macromolecular crystallography. This review provides an overview of this rapidly moving field in light of the rich dialogues and forward-thinking proposals explored during the congress workshop and microsymposium. These advances in microcrystallography shed light on the potential to reshape current research paradigms and enhance our comprehension of biological mechanisms at the molecular scale.

## Abstract 2

The 26th IUCr congress held in Melbourne brought discussions on micro-crystallization and *in vivo* crystallography within structural biology to the forefront, highlighting innovative approaches and collaborative efforts to advance macromolecular research.

## 1. Introduction

The intricate world of macromolecular structures has long been a field of extensive research and technological innovation. Reflecting on the dynamic evolution of the field, the recent advancements in micro-crystallization and *in vivo* crystallography stand at the forefront of structural biology. These methodologies offer unique insights into the atomic structures of macromolecules (Koopmann *et al.*, 2012 ▸). The quest to unravel the mysteries of macromolecular structures often hinges on the laborious task of crystal production. Historically, this endeavour necessitated crystals of substantial size to achieve diffraction profiles of adequate quality, representing a significant hurdle in structural biology (Schönherr *et al.*, 2015 ▸). However, the landscape of crystallography is undergoing a transformative shift, thanks to recent techno­logical advancements in sample handling within biological laboratories and synchrotron facilities (Duszenko *et al.*, 2015 ▸). These innovations now permit the use of markedly smaller crystals, significantly reducing the workload associated with their production. Nonetheless, mastering these techniques and fully leveraging the potential of microcrystals remains a formidable challenge, necessitating ongoing efforts to refine and democratize these methodologies (Russo Krauss *et al.*, 2013 ▸). This period of innovation and exploration is marked by an increased focus on developing the field, showcasing the critical role of cutting-edge research and collaborative exchanges in shaping the future of structural biology. This paradigm shift not only lightens the experimental load but also opens new vistas for exploration in structural biology, marking a pivotal moment in our pursuit of understanding biological processes at the atomic level (Martin-Garcia *et al.*, 2016 ▸).

*In vivo* crystallography, a pioneering field rediscovered for modern structural biology, traces its origins back to the early observations made in the late 19th century. Initially overlooked due to the minuscule sizes of naturally occurring crystals within living cells and the technical challenges associated with handling such small specimens, this domain lay dormant for decades. The resurgence of interest and advancements in this area greatly concern the revolutionary developments in sample characterization, identification and handling techniques at X-ray diffraction facilities (Gallat *et al.*, 2014 ▸). These technological leaps allowed proof-of-concept studies meant that structures could be determined from *in vivo* grown crystals despite their micrometre size, even when experimental phasing was required (Coulibaly *et al.*, 2007 ▸). Together, these advances have breathed new life into *in vivo* crystallography, transforming it into a vibrant and promising field of research that promises to unlock new understanding of biological molecules in their native, physiological contexts (Redecke *et al.*, 2013 ▸). Amidst these developments, the microsymposium and workshop organized at the International Union of Crystallography (IUCr) Congress served as a pivotal platform for discussing the rapid advancements and collaborative efforts that drive the field forward. Su *et al.* (2015 ▸) traced the transition of crystallography from its roots in mineralogy and mathematics to a pivotal tool in structural biology, driven by several key innovations. These include improvements in X-ray crystallography, the adoption of cryo-cooling techniques to radiation damage, enhanced imaging through synchrotron radiation and refined computational strategies for structure determination. Developments in microfluidics and the automation of crystal growth and screening processes have also been crucial, making the study of complex macromolecules more accessible. Furthermore, Shi (2014 ▸) reflected on the impact of X-ray crystallography on our understanding of biological processes, emphasizing its central role in structural biology (Shi, 2014 ▸). The narrative of development in the field, including unresolved challenges and the steady progress of crystallography, is detailed by Voytekhovsky (2019 ▸), highlighting the evolution of the discipline and its impact on science. Finally, Lifshitz (2002 ▸) discussed the discovery of quasicrystals, marking a significant rejuvenation in the field.

The organization of a microsymposium complemented by a dedicated workshop at the recent IUCr congress epitomizes the continuous effort to advance microcrystallography and *in vivo* crystallography. Such forums are instrumental in addressing the complexities spanning crystal production and identification, sample handling, and data acquisition at X-ray diffraction facilities, to sophisticated data analysis techniques. Esteemed figures and leading researchers in the field often convene to share their latest research and methodologies, fostering a collaborative environment that propels the scientific community forward. The dialogues and presentations at these gatherings not only present the current state of play but also chart out the path for future developments required for the field to expand. This article aims to capture the spirit of the discussions held during such pivotal events, providing insight into the promising directions and emerging inquiries poised to revolutionize our comprehension of macromolecular structures in forthcoming years.

## 2. Background

The evolution of macromolecular crystallography has been marked by significant milestones since the seminal work of Perutz *et al.* (1960 ▸) and Kendrew *et al.* (1958 ▸) in the 1950s, who laid the foundational stones by determining the structures of hemoglobin and myoglobin, respectively. This pivotal era heralded a new phase in structural biology, utilizing X-ray crystallography to elucidate the 3D structures of proteins at atomic resolution. Since then, technological and method­ological advancements have led to versatile approaches with growing adaptability to resolve the structures of ever more challenging targets (Rathore *et al.*, 2021 ▸). The development of synchrotron radiation sources in the 1970s and 1980s drastically improved the quality and speed of data collection, while the 1990s saw the advent of cryo-crystallography, which significantly reduced radiation damage to protein crystals during X-ray exposure [reviewed in Brändén & Neutze (2021 ▸)]. These milestones illustrate the journey towards the high-throughput structure determination that underpinned the emergence of structural genomics and rationale drug discovery.

Concurrently, computational tools for data processing and structure determination became more sophisticated, facilitating the analysis of increasingly complex macromolecules. The introduction of automation in crystal growth and screening has further streamlined the crystallization process, enhancing the efficiency of structural determination. These collective advances have expanded the scope of macromolecular crystallography, enabling researchers to tackle more challenging projects, such as the study of large protein complexes and membrane proteins, thus continuing to deepen our understanding of biological mechanisms at the molecular level (Thompson *et al.*, 2020 ▸). In this context, the advent of microcrystallography was initially driven by the difficulty in obtaining large crystals for targets with conformational heterogeneity or limited quantity. However, it has now emerged as a preferred route in many instances with the promise of improved speed and new applications in time-resolved crystallography beyond single-crystal approaches.

The challenges posed by membrane proteins, large protein complexes and proteins purified from natural sources have indeed fuelled the development of microcrystallography as well as complementary approaches such as cryo-electron microscopy (cryo-EM). This development represents a critical response to the intricate demands of modern biological research, marking a significant methodological shift. These biomolecules, crucial for a multitude of biological processes, often present difficulties in crystallization due to their size, compositional and conformational heterogeneity, and the hydro­phobic nature of membrane proteins. Traditional crystallography techniques, which require relatively large and well ordered crystals, frequently fall short when applied to these targets (Boudes *et al.*, 2014 ▸). Microcrystallography emerged as a solution, allowing for the structural analysis of microcrystals that are too small to be studied effectively by conventional means. This advancement was made possible through the integration of highly focused and high-flux X-ray beams available at synchrotron facilities, and coupled with fast, sensitive photo-counting detectors capable of capturing high-resolution data from these weakly diffracting crystals (Broennimann *et al.*, 2006 ▸; Yamamoto *et al.*, 2017 ▸). The advent of microcrystallography has been pivotal in extending the boundaries of structural biology, allowing for detailed insights into the complex structures of membrane proteins and large protein complexes, thereby catalysing breakthroughs in drug discovery and unravelling the intricate mechanisms of fundamental biological processes (Gallenito & Gonen, 2022 ▸). In these respects, it is similar to cryo-EM which has undergone a ‘resolution revolution’ in the last decade, accelerating the rate of structure determination of targets intractable for crystallization (Kühlbrandt, 2014 ▸).

Techniques such as serial femtosecond crystallography (SFX) and micro-electron diffraction (MicroED) have emerged as potent alternatives to synchrotron-based structural determination, particularly suited to the smallest microcrystals and even nanocrystals. SFX, leveraging the intense, ultra-short pulses of X-ray free-electron lasers (XFELs), allows the analysis of crystals far smaller than those required for conventional crystallography for the capture of diffraction data without significant radiation damage (Johansson *et al.*, 2012 ▸). This method is particularly beneficial for studying proteins that can only form micro- or nanoscale crystals, providing a pathway to elucidate previously intractable structures. On the other hand, MicroED utilizes transmission electron microscopes to obtain high-resolution diffraction data from nanocrystals, applying well established diffraction methodologies pioneered for X-rays but using commonly available electron microscopes. This technique has proven especially useful for determining the structures of small organic molecules, peptides and proteins with minimal sample quantities (Danelius & Gonen, 2021 ▸).

Building on innovative methodologies such as SFX and MicroED, *in vivo* crystallography introduces a novel dimension to structural biology by exploring the spontaneous crystallization of proteins and other macromolecules within the complex milieu of living cells. This approach permits an unprecedented glimpse into the dynamic processes of life at the molecular level, offering a window into biological mechanisms as they unfold under conditions that closely mimic their natural state. This link to cellular biology mirrors the potential of cryo-electron tomography (cryo-ET) to provide high-resolution structural information at the level of individual molecules in the context of the cell (Young & Villa, 2023 ▸). Through the lens of *in vivo* crystallography, scientists gain invaluable insights into the natural orchestration of crystal formation in organisms, shedding light on disease pathology and cellular functions with the potential to revolutionize biomedical research and our understanding of macromolecular self-assembly (Gallat *et al.*, 2014 ▸).

## 3. Workshop and microsymposium overview

**Figure 1:**
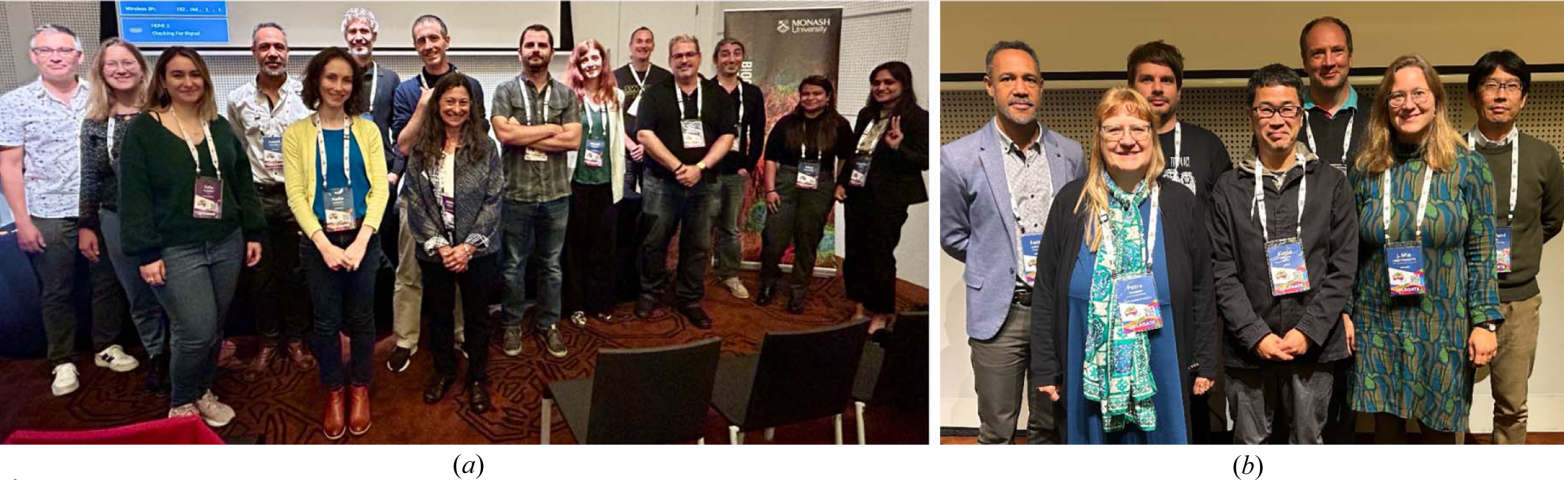
IUCr 2023 speakers in the X-ray microcrystallography workshop and the *in vivo* crystallography microsymposium. (*a*) Workshop (left to right): C. Sauter, M. Lahey-Rudolph, S. Trampari, F. Coulibaly, N. Zatsepin, D. Garriga, L. Chavas, J. Frank, R. Kirian, E. Campbell, T. Caradoc-Davies, T. Gonen, D. De Sanctis, L. Klecha and S. Ghosh. (*b*) Microsymposium (left to right): F. Coulibaly, P. Fromme, D. Oberthuer, K. Hirata, L. Redeke, M. Lahey-Rudolph and T. UeNo.

The microsymposium and workshop brought together leading scientists to discuss the latest developments in microcrystallography and *in vivo* crystallography (Fig. 1 ▸). The events were divided into sessions focused on the production, detection and handling of microcrystals, alongside serial approaches for sample presentation and data collection. Key presentations highlighted the structural biology of bacterial insecticides, advancements in protein crystallography using nanosized crystals, and innovative techniques for finding and diffracting protein crystals in living cells.

### 3.1. Workshop on advances in macromolecular microcrystallography: innovations in production, detection, handling and data collection of microcrystals

The workshop on ‘advances in macromolecule microcrystallography’ was introduced by F. Coulibaly from Monash University who highlighted the diversity of participants from across the globe, including Africa, which is the last continent lacking a microfocus beamline. The workshop demos and presentations reflected such worldwide interest in microcrystallography with contributions from researchers working across four continents [Fig. 1 ▸(*a*)].

**Figure 2:**
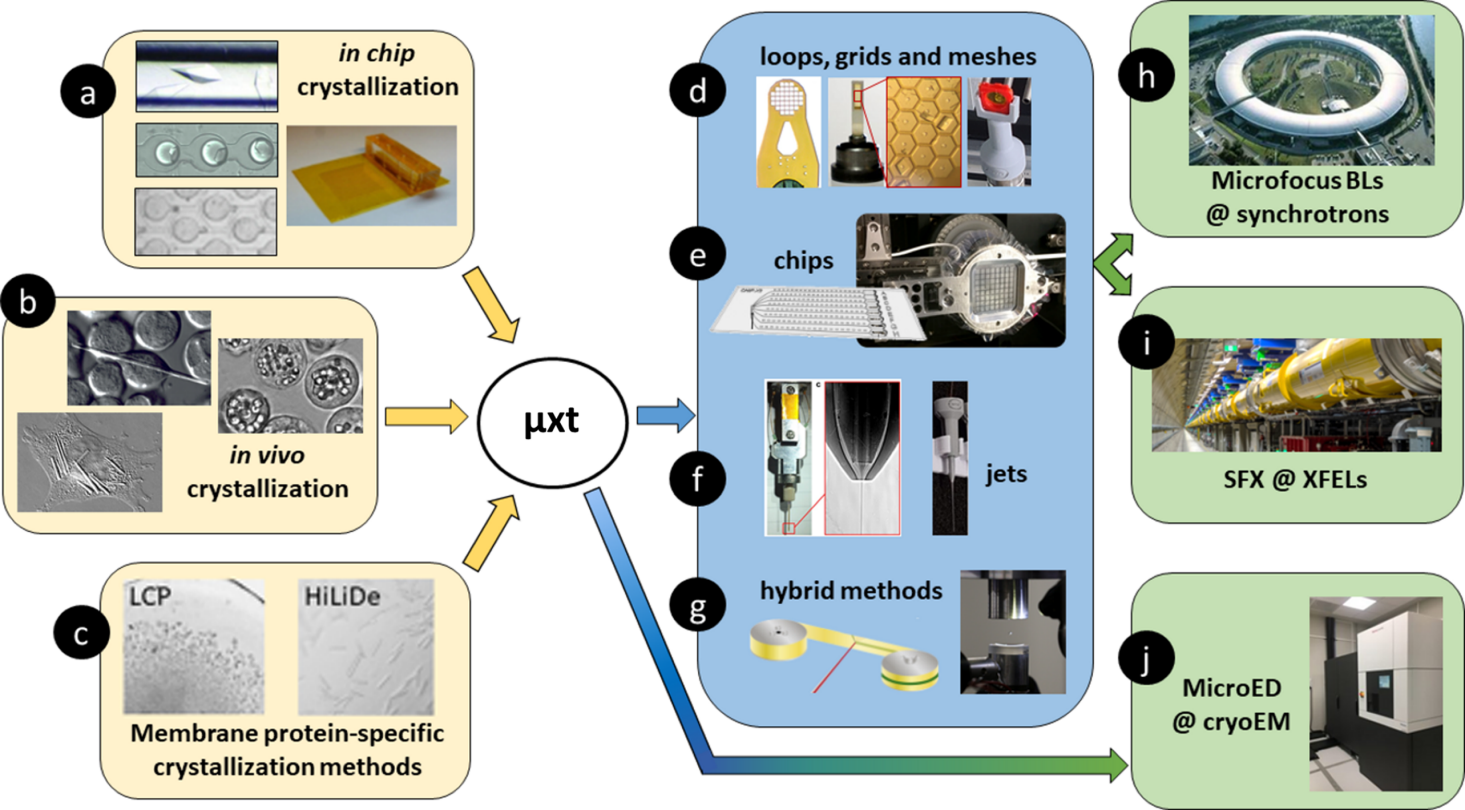
Specific methodologies underpinning microcrystallography (µxt). (*a*)–(*c*) Crystallogenesis requires approaches that differ from ‘traditional’ methods, including (*a*) in-chip and (*b*) in-cell crystallization, or (*c*) in-LCP crystallization and the HiLiDe method for membrane proteins. Presentation of the crystals to an X-ray beam requires specific approaches using (*d*) meshes, grids and small supports; (*e*) large-area chips; (*f*) jets; (*g*) or other combined technologies. (*h*)–(*j*) Diffraction experiments are possible thanks to the development of microfocus beamlines at synchrotrons and free-electron laser facilities across the world. (*j*) MicroED emerges as an alternative to X-ray diffraction for the smallest microcrystals. Figure adapted from the literature (Gicquel *et al.*, 2018 ▸; Heymann *et al.*, 2014 ▸; De Wijn *et al.*, 2019 ▸; Schönherr *et al.*, 2015 ▸; Manji & Friesen, 2001 ▸; Hasegawa *et al.*, 2011 ▸; Båth, 2022 ▸; Trampari *et al.*, 2021 ▸; Illava *et al.*, 2021 ▸; Owen *et al.*, 2017 ▸; Oberthuer *et al.*, 2017 ▸; Beyerlein *et al.*, 2017 ▸; Tsujino & Tomizaki, 2016 ▸) with permission. Images in (*d*) and (*f*) were provided by MiTeGen and SerialX, respectively.

During the workshop, participants were introduced to the latest innovations and developments in the field, reflecting more than three decades of progress since the inception of microcrystallography. The workshop highlighted the central role of dedicated microfocus beamlines now available at synchrotron sources across the world, capable of producing brilliant X-ray beams with diameters ranging from 1 to 10 µm. It then moved into the more recent advent of serial femto­second crystallography at XFEL facilities alongside the resurgence of MicroED in cryo-EM (Fig. 2 ▸). Challenges unique to microcrystallography, such as the detection, handling and data collection from micro- and nanocrystals, were addressed through innovative solutions designed to overcome issues like crystal fragility, susceptibility to radiation damage and the complexities of mounting these nearly invisible crystals for diffraction experiments. The workshop also explored a variety of strategies for presenting microcrystals to the X-ray beam and the corresponding data collection and processing techniques. With an emphasis on optimizing these methods and moving towards standardization, the event fostered discussions among scientists developing or applying these techniques, offering short talks, demos, hands-on sessions and a round-table discussion to contemplate future needs and trends in microcrystallography, thereby paving the way for broader accessibility and application in non-expert laboratories.

#### 3.1.1. Not your everyday crystal: creative methods in crystallogenesis

The first session featured four talks that collectively covered innovation and alternative methods in crystallogenesis [Figs. 2 ▸(*a*)–2 ▸(*c*)]. Claude Sauter, from the IBMC at the University of Strasbourg, kicked off with an overview of the current advancements in crystallogenesis, shedding light on foundational concepts and recent breakthroughs in macromolecular crystal formation. He insisted that the ‘phase diagram is your best friend’, which can now be delineated more precisely and conveniently using in-drop dynamic light scattering monitoring of nucleation and precipitation in crystallization experiments (Oberthuer *et al.*, 2012 ▸; De Wijn *et al.*, 2020 ▸). In recalcitrant cases, he discussed how molecular glues can act as nucleants to expand the crystallization space and increase success (Engilberge *et al.*, 2017 ▸).

The narrative then transitioned into the fascinating world of *in vivo* microcrystals, tracing its historical roots and discussing methodologies for *in situ* microcrystallography [Fig. 2 ▸(*b*)]. This segment was jointly presented by L. Chavas from the University of Nagoya and D. Garriga from the ALBA Synchrotron, who provided insights into the evolution and current practices within the field. They highlighted, respectively, the *iv*MX integrated pipeline at Nagoya University, and the possibility to solve structures directly within the cell without extraction of the intracellular crystals and even in-cell experimental phasing if required (Boudes *et al.*, 2017 ▸).

M. Lahey-Rudolph from the Technical University of Applied Sciences in Lübeck took the stage next, delving into the detection and analysis of *in vivo* microcrystals produced in insect cells by recombinant proteins expressed with a baculo­virus expression system. She presented the use of SAXS at synchrotron sources and fluorescent-assisted cell sorting for the detection of intracellular crystals in cases where as few as 0.3% of cells contain crystals (Lahey-Rudolph *et al.*, 2020 ▸, 2021 ▸; Schönherr *et al.*, 2024 ▸). The session ended with S. Trampari from DECTRIS Ltd, who navigated through the challenges and intricacies of membrane protein crystallization, identifying key bottlenecks encountered in the process. She presented the High Lipid High Detergent (HiLiDe) approach that offers a simple method to obtain crystals of membrane protein for cases where *in surfo* or *in meso* crystal forms are preferred (Trampari *et al.*, 2021 ▸) [Fig. 2 ▸(*c*)].

This comprehensive exploration of crystallogenesis set a solid foundation for the ensuing discussions, seamlessly transitioning to the second session, which delved deeper into the techniques for presenting microcrystals to the beam.

#### 3.1.2. Bring it on! Presentation of microcrystals to the beam

In the second workshop session, participants were treated to an in-depth exploration of innovative techniques for microcrystal presentation at synchrotron sources [Figs. 2 ▸(*d*)–2 ▸(*g*)]. R. Kirian from Arizona State University initiated the discussion with a talk on advanced liquid jet injectors for microcrystals that can be easily 3D printed (Konold *et al.*, 2023 ▸). He presented work in collaboration with A. Ros’ laboratory on interrupted liquid jets, where oil and crystal-containing droplets are interleaved in phase with the X-ray beam to use as little material as possible. The ∼100 folds in efficiency from the systems discussed can be particularly important at compact XFEL sources that are less efficient than femtosecond XFELs (Sonker *et al.*, 2022 ▸).

As an alternative mode for the delivery of microcrystals to the beam, C. Sauter delved into the realm of microfluidic chips, discussing a counter-diffusion design called ChipX with an optimized material allowing clear visualization and *in situ* diffraction [Fig. 2 ▸(*e*)]. The ability to add chemicals raised the prospects of lab-on-chip and ligand screening approaches using such devices (De Wijn *et al.*, 2019 ▸). The session transitioned to a practical, hands-on experience led by experts in the field. E. Campbell from the Australian Synchrotron provided a comprehensive overview of existing microcrystallography at the facility and examples of the serial crystallography capabilities that will be available at the new MX3 beamline to be commissioned in 2024. S. Ghosh from Nagoya University introduced specialized devices crafted for the meticulous handling of microcrystals [Figs. 2 ▸(*d*) and 2 ▸(*f*)]. J. Frank from MiTeGen completed the practical session by demonstrating the nuanced process of microcrystal harvesting, including the use of a humidity-controlled glovebox designed for the safe and efficient freezing of crystals [Fig. 2 ▸(*d*)].

This session not only highlighted the technological advances in presenting microcrystals to the beam but also emphasized the collaborative spirit of the scientific community in refining and sharing methodologies. As attendees absorbed these insights, the stage was set for the forthcoming discussions on X-ray beamline facilities, the processing of serial diffraction data and the application of MicroED, signalling a deeper dive into the technical aspects of crystallography.

#### 3.1.3. It takes all sorts. Three approaches to microcrystal diffraction

The third session of the workshop presented a trio of talks that spanned the spectrum of cutting-edge diffraction techniques [Fig. 2 ▸(*h*)–2 ▸(*j*)]. D. De Sanctis from ESRF opened the session with an insightful discussion of data collection at beamline ID29 in the ultra-low emittance fourth-generation synchrotron, delving into the intricacies of processing pipelines tailored for serial synchrotron data collection. The setup allows analysis of light-sensitive samples such as photoactivable cage compounds and experiments under anaerobic conditions, which can be key for enzymatic processes in the microsecond to millisecond range. This was followed by N. Zatsepin from La Trobe University, who provided a comprehensive introduction to serial crystallography data analysis at synchrotron and XFEL beamlines, emphasizing the utility of the *CrystFEL* software suite (White *et al.*, 2012 ▸) and *DatView* for visualizing and querying large datasets (Stander *et al.*, 2019 ▸). The session was brought to a compelling close by T. Gonen from the David Geffen School of Medicine at UCLA, who skilfully condensed two decades of progress in MicroED into an impressive 20 min summary spanning drug discovery, small-molecule forensics and membrane protein structures (Danelius *et al.*, 2023 ▸). This methodology offers unprecedented speed in the field of chemistry with the ability to go from ‘powder to structure’ within minutes from tiny amounts of chemicals even within a mixture (Jones *et al.*, 2018 ▸). Pushing the boundaries in protein crystallography, T. Gonen showed how a MicroED structure can be determined from a nanocrystal made up of only ∼1000 proteins. MicroED is not only applicable to very diverse fields but is also readily accessible experimentally with a global availability of cryo-electron microscopes that could be adapted to collect MicroED data. Thus, the conditions are here for an expansion of a methodology that has so far remained under-utilized.

The exceptional quality of the presentations underscored the dynamic and evolving nature of microcrystal diffraction methodologies, captivating the attendees with their depth and breadth. The session was a testament to the vibrant diversity and innovation within the field of crystallography, setting the stage for the conclusive segment of the workshop. Following these enlightening discussions, the workshop culminated in a round-table dialogue involving all speakers and the audience. This final gathering provided valuable perspectives on the future trajectory of microcrystallography, fostering a vision for its advancement and the challenges that lie ahead. Building on the rich discussion, future developments in microcrystallography will crucially hinge on improving crystal detection and ease of handling, potentially through advancements in microfluidics. Leveraging the combined power of synchrotron radiation, XFELs and MicroED is poised to significantly broaden the applicability of the technique. Although access to specialized synchrotron instruments remains a bottleneck, rapid technological progress is expected to alleviate this challenge. Additionally, emerging AI-based tools are set to enhance crystal quality and could revolutionize methods for crystal production, marking a new era of innovation in structural biology (Li *et al.*, 2023 ▸; Sumida *et al.*, 2024 ▸; Huddy *et al.*, 2024 ▸).

### 3.2. Microsymposium on *in vivo* crystallography and synchrotron radiation

Aligned with the workshop, this microsymposium illuminated the fascinating world of natural crystal growth within living organisms and cells, a phenomenon known for over a century but only recently understood to be pervasive across all kingdoms of life. Initially perceived as oddities, these *in vivo* crystalline formations are now recognized for their diverse functional roles, from energy storage and viral persistence to self-defence mechanisms. This symposium aimed to spotlight the advancements in harnessing these phenomena for the structural analysis of macromolecules directly within their native environments. Showcasing research efforts from France, Germany, Australia, Japan and the USA, the event highlighted integrated solutions, and national and international projects that promise to revolutionize traditional methods of structure determination [Fig. 1 ▸(*b*)]. Emphasizing the critical role of direct access to advanced synchrotron instruments, the symposium explored the comprehensive application of synchrotron radiation – from soft X-ray transmission cryo-microscopy to X-ray diffraction – in characterizing these natural occurrences. The discussions extended to the potential of XFEL facilities and advanced micrometre-sized synchrotron X-ray beams in unveiling the 3D structures of *in vivo*-grown crystals. Gathering experts who have developed and applied innovative instrumentation and methodologies, the microsymposium underscored the imperative of an integrated approach at synchrotron facilities to fully unlock and leverage the mysteries of *in vivo* crystallography.

The microsymposium on ‘*In Vivo* Crystallography and Synchrotron Radiation’ featured a series of six talks that collectively showcased the cutting-edge of structural biology and crystallography within living systems. D. Oberthuer from CFEL in Hamburg delved into the structural biology of bacterial insecticides, elucidating the use of *in vivo* grown crystals and nano-scale serial femtosecond crystallography (nanoSFX) to explore their mechanisms (Williamson *et al.*, 2023 ▸). K. Hirata from SPring-8 in Japan showcased the cutting-edge approach to protein crystallography including a ‘hyper focused beam’ that allows structure determination from extremely small, 500 nm-sized crystals. This breakthrough was facilitated by the superior quality of state-of-the-art X-ray diffraction sources, alongside sophisticated data collection and processing pipelines (Hirata *et al.*, 2019 ▸). These advancements have expanded the limits of structural analysis, enabling researchers to work with crystals at the frontier of size limitations, thus opening new avenues for detailed molecular exploration and understanding.

P. Fromme from Arizona State University (ASU) significantly advanced the discussion by delving into the realm of time-resolved femtosecond crystallography. Her presentation illuminated the audience on how new technological strides, particularly the development of a compact free-electron laser (CFEL), could revolutionize the study of biomolecular dynamics. These advancements, being pioneered at ASU (Graves *et al.*, 2018 ▸), are crucial for facilitating time-resolved studies on small crystals. This marks a significant leap forward in our capability to observe and understand the rapid, intricate processes that govern biomolecules in real time. Importantly, the CFEL is designed as an institute-based X-ray source with the potential to democratize a methodology that to date suffers from a high-barrier to access.

**Figure 3:**
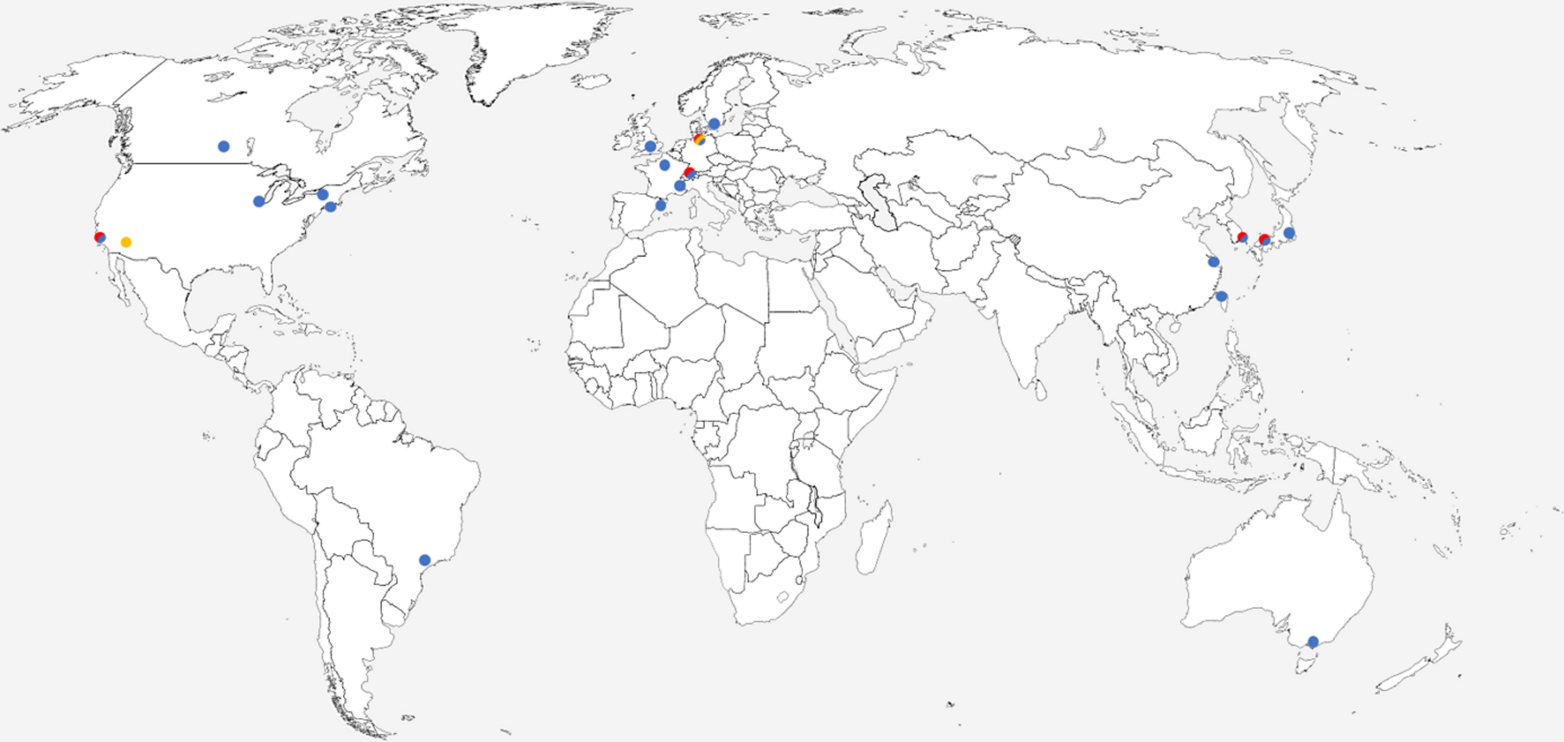
Worldwide distribution of X-ray based facilities where data from microcrystals can be collected. Facilities hosting microfocus MX beamlines (blue dots), XFELs (red dots) and compact FELs (yellow) are indicated on the map. In addition, many facilities across the world host cryo-electron microscopes capable of collecting MicroED data from microcrystals (not indicated on the map).

J. M. Lahey-Rudolph offered a fascinating exploration into identifying and diffracting protein crystals within living insect cells. In this instance, she presented a fixed-target approach at LCLS to determine the structures of intracellular crystals from cells spread on micro-patterned silicon chips with only 15 min of beam time (Lahey-Rudolph *et al.*, 2021 ▸). Also from Lübeck University, L. Redecke not only discussed the challenges and successes of intracellular protein crystallization, but also introduced the *InCellCryst platform* (Schönherr *et al.*, 2024 ▸). This platform is based on the recombinant expression of proteins in High Five insect cells using the baculovirus expressions system. To target the proteins to specific cellular sub-compartments that may enhance crystallization, the proteins are genetically modified to include addressing tags, short peptide sequences that direct the proteins to designated cellular locations. This strategic localization is crucial for optimizing the conditions that favour protein crystallization within the cell. In addition, a method was introduced to enhance the sizes of naturally occurring small crystals by aggregating cells and inducing cell-to-cell fusion, which facilitates the growth of larger crystals. This approach maintains crystals in a cellular environment but makes *in vivo* crystallography more accessible to researchers who are not accustomed to working with microsamples. If it can be generalized, this advancement would open up *in vivo* crystallography to a broader scientific community by enhancing the feasibility of using conventional synchrotron sources for this purpose. Though many modern synchrotrons are equipped with microfocus beamlines (Fig. 3 ▸, Table 1 ▸), these beamlines are often oversubscribed, and typically require larger amounts of samples. A larger crystal size is more compatible with conventional beamlines, thereby easing access and reducing the dependency on specialized microfocus capabilities.

Finally, T. Ueno from the Tokyo Institute of Technology concluded the session with a presentation on cell-free and in-cell protein crystallization for high-throughput screening. Cell-free protein crystallization allows rapid screening in ∼3 days with a reaction volume of only tens of microlitres, showcasing the efficiency of the method (Abe *et al.*, 2022 ▸).

## 4. Conclusions

Drawing on the rich discussions and ground-breaking advancements presented throughout the microsymposium and workshop, our manuscript has journeyed through the evolving landscape of micro-crystallization and *in vivo* crystallography. We have witnessed the fusion of innovative technologies and methodologies, from revolutionary instrumentation enabling time-resolved studies on minuscule crystals to the implementation of dedicated platforms facilitating the growth of crystals within living cells for wider accessibility. As we look ahead, the challenge remains to further democratize these techniques. This is already underway with (i) published pipelines for the production of microcrystals *in vitro*, *in cell* or cell-free; (ii) simpler sample handling including commercially available chips and 3D-printable jet injectors; (iii) the multiplication of sources for diffraction experiment-based XFELs (Fig. 3 ▸, Table 1 ▸) and widely available electron microscopes for MicroED; and (iv) more user-friendly computational approaches to broaden the impact of microcrystallography. Embracing these challenges will propel us toward unravelling the complex biological mechanisms at the heart of structural biology.

Cryo-EM has become a broadly available alternative for structure determination of difficult targets under near-native or – in the case of cryo-ET – native conditions. Technological and methodological advances largely bridged the gap with classical crystallography. Yet, they remain complementary to advanced crystallography methods such as microcrystallography which offers high resolution and throughput, and unparalleled opportunities for time-resolved studies.

From this review, a clear path emerges towards overcoming the limitations of traditional crystallography and unlocking the full potential of microcrystallography for answering fundamental biological questions.

## Figures and Tables

**Table 1:** X-ray based facilities where data from microcrystals can be collected Current lists for all synchrotrons can be found here, but do not specify microfocus capabilities: https://lightsources.org/lightsources-of-the-world/ and https://en.wikipedia.org/wiki/List_of_synchrotron_radiation_facilities.

Beamline	Country	Microfocus availability	Focused beam size, *H* × *V* FWHM (µm)	Aperture-, CRL- or collimator-defined beam size	Flux at 12–13 keV (photons s^−1^)	Energy (keV)
APS, 14-ID-B	United States		20 × 15		7 × 10^13^	8.5–15
APS, 17-ID-B	United States	2010	70 × 30	5, 10, 20	7.7 × 10^12^	6.5–20
APS, 19-ID-D	United States		100 × 20	5, 10, 20		6.5–19
APS, 21-ID-D	United States		10 × 10		5 × 10^12^	6.5–20
APS, 22-ID-D	United States		30 × 20		1 × 10^13^	6–16
APS, 23-ID-B	United States	2009	80 × 22	5, 10, 20	6.4 × 10^12^	6–20
APS, 23-ID-D	United States	2009	25 × 22	5, 10, 20	9.9 × 10^12^	8.3–20
APS, 24-ID-C	United States		60 × 20	5 to 70	1 × 10^12^	6.5–20
APS, 24-ID-E	United States		120 × 20	5 to 70	5 × 10^12^	12.68
ALBA, XAIRA	Spain	Expected 2024	3 × 1	1 × 1 to 20 × 20	3.5 × 10^13^	4–15
Australian Synchrotron, MX2	Australia	2009	25 × 15	7.5, 10, 20	3.6 × 10^12^	8–15.5
Australian Synchrotron, MX3	Australia	Expected 2024	2 × 2	5	1 × 10^14^	10–15
CLS, CMCF-BM	Canada		200	20, 50, 100, 150	1 × 10^11^	6–19
CLS, CMCF-ID	Canada	2010	53.6 × 8.6	5, 10, 20, 30, 50	1 × 10^13^	5–20
CHESS, FlexX	United States	2004	100	9 × 12	10^12^	8–14
DLS, I02-1 VMXm	United Kingdom	2018	0.4 × 1.3 to 9 × 13			10–22
DLS, I02-2 VMXi	United Kingdom	2017	10 × 10		5 × 10^13^	16
DLS, I03	United Kingdom	2006	90 × 20	20, 50, 100	1 × 10^13^	5.5–25
DLS, I04	United Kingdom	2006	110 × 100	10 × 5	1.25 × 10^12^	6–18
DLS, I04-I	United Kingdom	2006	60 × 50	10, 20, 30, 50, 70	3.8 × 10^12^	13.5
DLS, I24	United Kingdom	2008	7 × 6 to 50 × 50	5 × 5	5 × 10^12^	7–25
ESRF, ID23-1	France	2005	45 × 30	10, 20	1–4 × 10^12^	6–20
ESRF, ID23-2	France	2022	8 × 25	1 × 2	1.5 × 10^13^	14.2
ESRF, ID29	France	2022	0.5 × 0.5 to 6 × 5		10^16^	10–25
ESRF, ID30B	France	2015	200 × 200	20 × 20	6 × 10^12^	6–20
ESRF, MASSIF-1	France	2015	100 × 100	10 × 10	5 × 10^12^	12.65
ESRF, MASSIF-3	France	2014	15 × 15		2 × 10^13^	12.81
LNLS, Manacá	Brazil	2020	0.5 × 0.5 and 10 × 7		10^13^	5–20
MAX IV, BIOMAX	Sweden	2017	100 × 100	5, 10, 20, 50, 100	5 × 10^12^	6–24
MAX IV, MicroMAX	Sweden	2023	5 × 5		10^14^	5–25
NSLS II, AMX	United States	2017	7.5 × 5		4.3 × 10^12^	9.5–18
NSLS II, FMX	United States	2017	10 × 10	1 × 1.5	4 × 10^12^	5–30
PETRA III, P11	Germany	2013	9 × 4	1 × 1	1 × 10^13^	5.5–30
PETRA III, P13	Germany	2014	30 × 20	10	5 × 10^12^	4.5–17.5
PETRA III, P14	Germany	2014	6 × 2		2 × 10^13^	7–20
Photon Factory, BL1-A	Japan	2010	13 × 13		1.4 × 10^11^	3.8–4.6, 6.5–7.3, 11.6–12.9
Photon Factory, BL17-A	Japan	2006	40 × 20	10, 20, 40	2.1 × 10^11^	5.6–13.8
PAL, 11C	Korea	2017	8.5 × 4.1		1.3 × 10^12^	5–20
SSRF, BL17U1	China		67 × 23		4.1 × 10^12^	5–18
SSRF, BL18U1	China		10 × 7		5 × 10^11^	5–18
SOLEIL, PX1-A	France	2013	40 × 20		2 × 10^12^	6–15
SOLEIL, PX2-A	France	2013	10 × 5	5	3.5 × 10^12^	6–18
SPring-8, BL32XU	Japan	2010	10 × 15	1 × 1	7 × 10^10^	9–15
SPring-8, BL41XU	Japan	2014	35 × 50	2 × 2	4.3 × 10^13^	6.5–17.7
SSRL, BL12-1	United States		200 × 150	5 × 40	4 × 10^12^	6.2–18
SSRL, BL12-2	United States	2009	150 × 150	15	4 × 10^12^	6.7–17.0
SLS, PXI X06SA	Switzerland	2004	100 × 100 to 5 × 5	10 × 1, 2 × 1	2 × 10^12^	5.7–17.5
SLS, PXII X10SA	Switzerland	2005	73 × 16	30 × 15, 10 × 10	2 × 10^12^	6–20
TPS, 05A	Taiwan	2018	65 × 36	20, 10	2 × 10^12^	5.7–20
TPS, 07A	Taiwan	2022	30 to 1		10^12^ to 10^13^	6–20
